# Monocytes from Chronic HBV Patients React *In Vitro* to HBsAg and TLR by Producing Cytokines Irrespective of Stage of Disease

**DOI:** 10.1371/journal.pone.0097006

**Published:** 2014-05-13

**Authors:** Arjan Boltjes, Zwier M. Groothuismink, Gertine W. van Oord, Harry L. A. Janssen, Andrea M. Woltman, André Boonstra

**Affiliations:** 1 Department of Gastroenterology and Hepatology, Erasmus MC University Medical Center, Rotterdam, The Netherlands; 2 Liver Clinic University Health Network, Division of Gastroenterology, University of Toronto, Toronto, ON, Canada; CRCL-INSERM, France

## Abstract

Individuals who are chronically infected with the hepatitis B virus (HBV) are highly heterogenous with respect to serum levels of HBV DNA, HBV particles and viral proteins. Since circulating leukocytes, such as monocytes, are constantly exposed to these viral components, it is likely that the functionality of these cells is affected. However, at present, little information is available on the consequences of the interaction between monocytes and viral components. Therefore, we examined the *in vitro* effects of HBV surface antigen (HBsAg) on monocytes and evaluated whether these effects were reflected *in vivo*. We observed that *in vitro* HBsAg exposure of monocytes induced robust production of IL-6 and TNF. However, between chronic HBV patients with distinct levels of serum HBsAg, HBV early antigen (HBeAg), and HBV DNA, TLR-induced monocyte cytokine production did not differ. Importantly, HBsAg-induced cytokine production by monocytes was similar between patients and healthy controls showing that earlier *in vivo* exposure to HBsAg does not affect the *in vitro* response. Additionally, we show that IL-10 is able to inhibit cytokine production by HBsAg-induced monocytes. In conclusion, we demonstrate that monocytes can recognize and respond to HBsAg, resulting in vigorous pro-inflammatory cytokine production *in vitro*. However, phenotype and function of the monocyte compartment in chronic HBV patients are not influenced by differences in levels of serum viral components, suggesting that regulatory mechanisms are active to avoid excessive *in vivo* monocyte activation.

## Introduction

Hepatitis B virus (HBV) infection is a major health problem. Although the majority of infected individuals clear the virus spontaneously, a fraction of patients is unable to clear the virus and develops a chronic form of hepatitis. Their numbers have already reached over 240 million people [1]. In time, persistence of HBV can lead to progressive liver damage, which increases the patient's risk of developing liver cirrhosis, liver failure and liver cancer. Chronicity of HBV is the result of a complex interaction between the replicating virus and an inadequate immune response [2]–[4]. After infection, viral replication takes place inside hepatocytes, and the secretion of infectious virions can take place for decades at high rates, and consequently HBV DNA, as well as viral proteins, like HBV early antigen (HBeAg) and HBV surface antigen (HBsAg), can be easily detected in serum. The levels of these clinical markers may fluctuate over time and are a reflection of disease activity and commonly used to define the patients' disease stage [3], [4].

Although circulating monocytes represent about 10% of leukocytes in human blood, relatively little is known on the consequences of chronic viral infections on monocytes. In HIV infections impaired monocyte functions have been reported [5], [6], and we recently demonstrated altered Toll-like receptor (TLR) responsiveness of monocytes obtained from patients with chronic HCV infections [7], [8]. Monocytes can be divided into two distinct subpopulations that are discerned based on their surface expression of CD14 and CD16. CD14^high^CD16^−^ monocytes make up the majority (80–90%) of blood monocytes, and have been reported to produce relatively high IL-10 and weak TNF levels, whereas the CD14^+^CD16^+^ subpopulation produces higher levels of pro-inflammatory cytokines, such as TNF and IL-1β [9], [10].

Also in chronic HBV, some studies reported modulation of the monocyte compartment as a result of the disease. Depending on the clinical phase of the chronic HBV infection altered monocyte subsets frequencies were reported [11], [12]. Moreover, PBMC from HBeAg-positive patients produced less TNF and IL-6 upon stimulation with TLR2 agonists as compared to HBeAg-negative patients [13], which was explained by lower expression of TLR2 in HBeAg-positive patients [14]. Furthermore, exposure of monocytes to HBsAg suppressed LPS-induced TNF and IL-1β production [15], while others reported that HBsAg has an immunostimulatory effect by inducing TNF and IL-10 production [16]. Since the consequences of constant exposure of peripheral monocytes to viral particles and the viral proteins HBeAg and HBsAg are still not completely understood, we here studied the *in vitro* and *in vivo* effects of these molecules on the phenotype and function of peripheral monocytes.

## Materials and Methods

### Patients and ethics statement

Peripheral blood was collected from patients chronically infected with HBV who visited the outpatient clinic of the Erasmus Medical Center. Patients eligible for the study were positive for HBsAg, and were not on-treatment before blood samples were taken for this study. Patients co-infected with human immunodeficiency virus, hepatitis A virus, hepatitis C virus or hepatitis D virus were excluded. Patient characteristics are presented in Table 1. The medical ethical committee of the Erasmus MC University Medical Center approved the study and all patients gave written informed consent before inclusion.

**Table 1:** Patient characteristics.

	Group 1	Group 2	Group 3
Number	14	20	11
Sex (F:M)	5∶9	11∶9	6∶5
Age	41.1 (24–59)^a^	35.1 (20–53)	31.4 (18–48)
ALT (IU/L)	83 (21–366)	37 (15–102)	111 (22–443)
Viral load (IU/mL)	63.8×10^6^ (28,300–6.0×10^8^)	1,767 (20–6,490)	1.0×10^9^ (29,200–2.6×10^9^)
HBeAg level (IU/mL)	7.96 (0–66.65)	0.17 (0–2.38)	944 (389–3,259)
HBsAg level (IU/mL)	10,341 (56–31,660)	3032 (108–17,448)	53,581 (542–125,820)

a Values for age, ALT, viral load, HBeAg level and HBsAg level are mean (minimum – maximum value).

### Laboratory measurements

HBsAg levels and HBeAg levels were measured in sera from a total of 45 chronic HBV patients using the Architect HBsAg assay (Abbott Laboratories, Abbott Park, IL, USA; range 0.05–250 IU/ml) or HBeAg assay (Abbott Laboratories; interpreted using a ratio of the sample relative light unit (RLU) rate to the cut-off RLU (S/CO)). HBV DNA levels were measured in serum using the Cobas TaqMan (Roche Diagnostics; lower limit of quantification, 20 IU/ml). ALT was measured as part of standard diagnostic procedures. HBV genotype was determined by means of the INNO-LiPA assay (Innogenetics, Gent, Belgium).

### Flow cytometric analysis of monocyte subpopulations

To determine the frequencies of monocyte subpopulations, whole blood was lysed and stained with antibodies against CD14 and CD16 (61D3 and 3G8, respectively; both eBioscience, San Diego, CA, USA), and measured by flow cytometry using a BD FACSCanto II (BD Biosciences, San Diego, CA, USA). Data was analyzed using FlowJo 7.6.5 software (Tree Star, Inc., Ashland, OR, USA).

### Monocyte stimulation and ELISA

Peripheral blood was collected from chronic HBV patients or healthy controls in sodium-heparin tubes, and PBMC were isolated by Ficoll-Paque (GE Healthcare, Uppsala, Sweden) gradient centrifugation, and frozen. PBMC (1×10^6^ cells/ml) were thawed and stimulated in 96-well plates in 250 µl X-VIVO culture medium (Lonza, Verviers Sprl, Belgium) containing penicillin/streptomycin (Gibco, Paisley, UK), L-glutamin (Lonza), and HEPES (Lonza) as described previously [7]: the cells were cultured either unstimulated or stimulated with 100 ng/ml Toll-like receptor (TLR) 2 ligand Pam3CSK4, 100 ng/ml TLR4 ligand LPS (both from Invivogen), 1 µg/ml TLR7/8 ligand R848 (Enzo Life Sciences, Antwerp, Belgium), or human plasma-derived pHBsAg Ay (American Research Products (ARP), Waltham, MA, USA) at a concentration of 5 µg/ml unless mentioned otherwise. In the experiment shown in Figure S1, in parallel to plasma-derived HBsAg Ay (5 µg/ml) stimulation, PBMC were also stimulated with recombinant HBsAg (1 µg/ml; Prospec, Rehovot, Israel) for 5 hours. In the IL-10 inhibition experiment, 10 ng/ml IL-10 (Miltenyi Biotec, Bergisch Gladbach, Germany) or medium as a negative control, was added at the same time as pHBsAg (1 µg/ml; ARP). After a total of 18 hours of culture, supernatants were harvested, and cytokine production (IL-6 and TNF) was determined by ELISA (all kits from eBioscience).

### Intracellular cytokine staining

The frequency of cytokine-producing monocytes was determined by measuring cytokines with intracellular cytokine staining using flow cytometry. PBMC were stimulated with TLR ligands or pHBsAg Ay (ARP) as described above, After culturing for 2 hours, brefeldin A (10 µg/ml; Sigma-Aldrich, St. Louis, MO, USA) was added to all wells. After 16 hours, the cells were harvested, incubated with LIVE/DEAD Fixable Aqua Dead Cell Stain (Invitrogen, Ltd., Paisley, United Kingdom), fixed with 2% formaldehyde, permeabilized with 0.5% saponin (VWR, West Chester, PA, USA) and stained for IL-6 (MQ2-13A5; eBioscience), and TNF (Mab11; eBioscience), and the surface markers CD14 and CD45 (61D3 and HI30, respectively; both eBioscience). Flow cytometric data was acquired and analyzed as described above.

### Statistical analysis

To compare clinical groups, groups were analyzed using the non-parametric Kruskal-Wallis test, followed by Dunn's multiple comparisons test. The frequencies of cytokine-producing cells determined at different HBsAg doses were normalized to the values obtained under the 1 µg/ml HBsAg condition. Differences between the cytokine expression of the healthy individuals and the group with chronic HBV patients were analyzed using the Mann-Whitney U test. A P-value of <0.05 was considered statistically significant. Graphpad Prism 5 was used for statistical analysis.

## Results

### HBsAg induces cytokine production by monocytes *in vitro*

**Figure 1:**
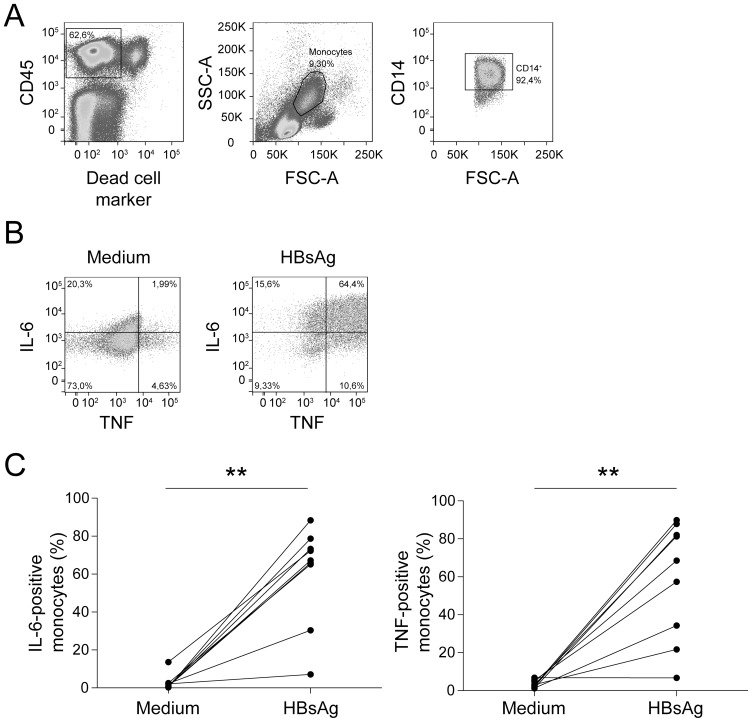
HBsAg induces cytokine production by monocytes. PBMC from healthy individuals were stimulated with HBsAg. After overnight incubation, cells were stained for CD14, IL-6 and TNF. (**A**) Gating strategy: Viable monocytes were identified on the basis of their forward-sideward scatter profile and their membrane expression of CD14. (**B**) Representative intracellular cytokine stainings are presented showing IL-6 and TNF-producing monocytes upon incubation with medium and HBsAg. (**C**) The frequencies of monocytes producing IL-6 or TNF upon incubation with medium or HBsAg are presented (n = 9, ** p<0.01, Wilcoxon signed rank test).

To investigate the modulatory effect of HBsAg on the functionality of blood monocytes, PBMC from healthy individuals were cultured in the presence of HBsAg. We compared monocytes, defined in Figure 1A as CD45 and CD14-expressing cells with their characteristic FSC and SSC profile, after overnight culture with either medium or HBsAg. As shown in Figure 1B and quantified in Figure 1C, the frequency of cytokine-producing monocytes was increased upon exposure to HBsAg *in vitro*. Upon exposure to HBsAg *in vitro*, the mean frequency of IL-6 and TNF-producing monocytes was increased from 2.6% to 61% and from 3.5% to 59%, respectively (Figure 1C). The percentages of monocytes producing IL-12p40, IL-15, and IL-10 were relatively low, while the chemokines CCL4 and CXCL8 were strongly induced (data not shown).

### The ratio of monocyte subpopulations is comparable in chronic HBV patients with different levels of HBV DNA or serum HBV protein levels

**Figure 2:**
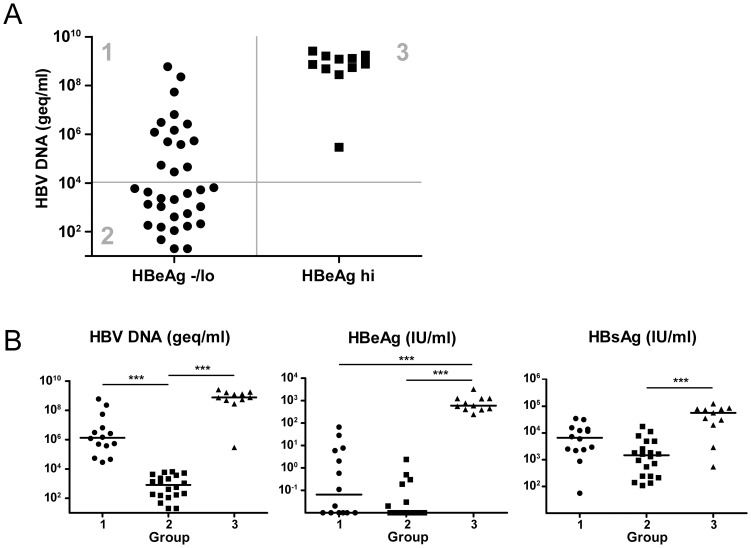
Chronic HBV patients divided into three groups based on HBV DNA and HBeAg levels. HBeAg levels, HBsAg levels and HBV DNA were measured in 45 chronic HBV patients. (**A**) HBV patients were divided into an HBeAg^negative^/HBeAg^low^ group (<100 IU/ml), and an HBeAg^high^ group (>100 IU/ml). Based on these two groups and HBV DNA levels, three groups of chronic HBV patients were defined (groups 1, 2 and 3). (**B**) Groups 1–3 were compared based on HBV DNA levels, HBeAg levels and HBsAg levels. *** p<0.0001, Kruskal-Wallis test, followed by Dunn's multiple comparison test.

The stimulatory effect of HBV proteins on blood monocytes upon exposure *in vitro* may have consequences for the functionality of the monocyte compartment in patients chronically infected with HBV. To examine this, we selected groups of chronic HBV patients differing in the levels of HBV DNA, HBeAg, and HBsAg. As shown in Figure 2A, three groups were identified based on HBV DNA and HBeAg levels. The experimental groups differed not only in the levels of HBV DNA and HBeAg, but also in HBsAg levels (Figure 2B). We defined group 1 as having low or undetectable HBeAg levels, and intermediate HBV DNA levels and HBsAg levels; group 2 as having low or undetectable HBeAg, and low HBV DNA and HBsAg levels; group 3 as having high levels of HBeAg, HBV DNA and HBsAg (Figure 2; Table 1). Patient groups did not differ with respect to age or gender.

**Figure 3:**
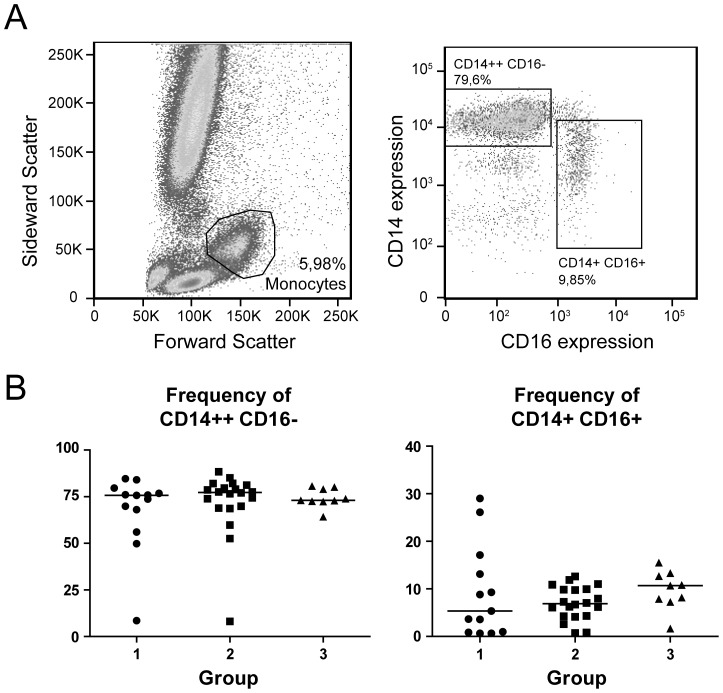
Different HBV DNA, HBeAg and HBsAg levels do not alter the frequency of monocyte subpopulations. Whole blood samples from chronic HBV patients were stained for CD14 and CD16 to analyze frequencies of the monocyte CD14^++^CD16^−^ and CD14^+^CD16^+^ subpopulations. (**A**) Gating strategy: monocytes were identified on the basis of their forward-sideward scatter, and divided into CD14^++^CD16^-^ and CD14^+^CD16^+^ populations. (**B**) The frequencies of monocyte subpopulations were compared between groups 1–3 (n = 45).

Monocytes can be divided on the basis of their expression of CD14 and CD16, and these subpopulations have been reported to exert distinct functions [9], [10]. To examine whether the chronic HBV patients in group 1, 2 and 3 differed in monocyte composition, we first determined the frequency of the monocytes expressing CD14 and CD16 in fresh peripheral blood samples from patients. As shown in Figure 3, the ratio of CD14^++^CD16^-^ and CD14^+^CD16^+^ monocytes did not differ between the distinct patient groups, indicating that chronic exposure to different amounts of HBV DNA, HBsAg and HBeAg did not lead to changes in the composition of the monocyte compartment.

### Monocyte function does not differ between HBV patient groups

**Figure 4:**
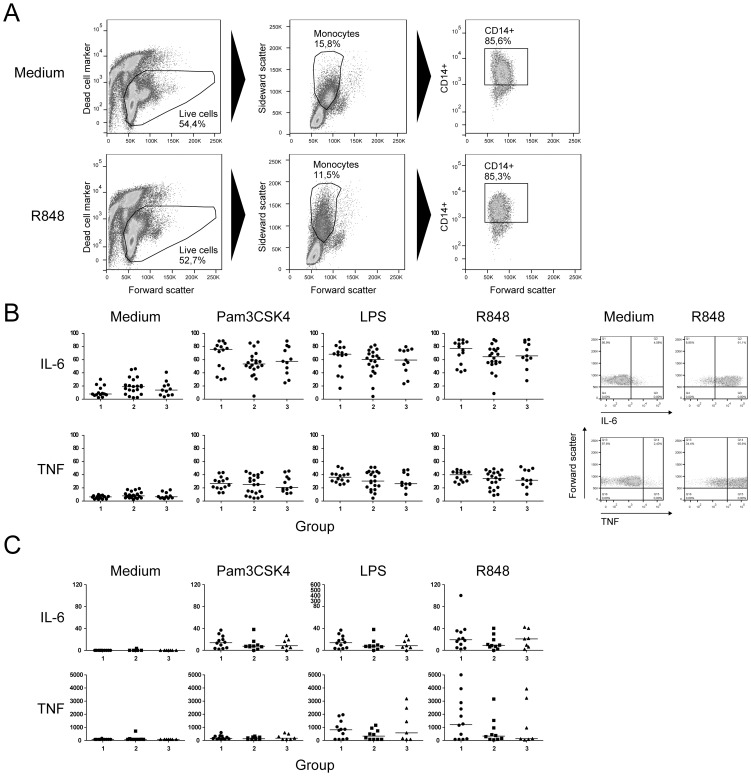
Monocytes from different patient groups display similar TLR-induced cytokine production. PBMC from patients with distinct HBV DNA, HBeAg and HBsAg profiles were incubated overnight with medium, Pam3CSK4, LPS or R848 to investigate the *in vivo* effect of HBV DNA levels, HBeAg levels and HBsAg levels on monocyte function. (**A**) Gating strategy: viable monocytes were identified on the basis of their forward-sideward scatter profile, and subsequently CD14^+^ monocytes were gated to assess cytokine production by intracellular cytokine staining (n = 45). (**B, left**) The frequencies of monocytes producing IL-6 and TNF upon medium or TLR ligand stimulation were compared between groups 1–3. (**B, right**) Representative dot-plots showing cytokine-positive cells induced by medium and R848 in one patient. (**C**) The cytokine levels measured in supernatant by ELISA (in ng/ml) upon stimulation of PBMC were compared between patient groups 1–3 (n = 30).

Since we showed that *in vitro* exposure of monocytes to HBsAg strongly induced the production of IL-6 and TNF, we explored whether the function of monocytes was affected by continuous exposure of monocytes to viral proteins in patients. To examine this, PBMC obtained from the patients with distinct serum HBV DNA, HBeAg and HBsAg levels were incubated overnight with medium or the TLR2 ligand Pam3CSK4, the TLR4 ligand LPS, or the TLR7/8 ligand R848, and cytokine production was measured by intracellular cytokine staining and ELISA. A high percentage of monocytes produced IL-6 and TNF after TLR stimulation (Figure 4A and 4B). As shown in figure 4B, TLR ligation of PBMC from chronic HBV patients with distinct serum levels of HBV DNA, HBeAg and HBsAg resulted in similar percentages of IL-6 and TNF producing monocytes. This was observed for agonists against TLR2, TLR4 and TLR7/8. Importantly, also the baseline frequencies of cytokine-producing monocytes were not different between the three patient groups, suggesting that circulating monocytes from patients were not at a higher activation state as evidenced by spontaneous cytokine secretion. Next, detailed data-analysis using SPICE software was performed to evaluate the simultaneous production of multiple cytokines by individual TLR-stimulated monocytes [17]. Again, no differences were observed when comparing the patient groups (data not shown). Besides frequencies, we also evaluated the intensity of the fluorescent signals representing the amounts of cytokines produced by monocytes upon stimulation, and also these parameters were not different between the patient groups (data not shown). In line with the above findings, also the amounts of TLR-induced IL-6 and TNF produced by monocytes, as measured by ELISA, were similar in the three groups of chronic HBV patients that differed in their serum levels of HBV DNA, HBsAg and HBeAg (Figure 4C).

### Monocytes obtained from patients exposed in vivo to distinct levels of HBV DNA, HBeAg and HBsAg, react similarly to HBsAg stimulation *in vitro*

**Figure 5:**
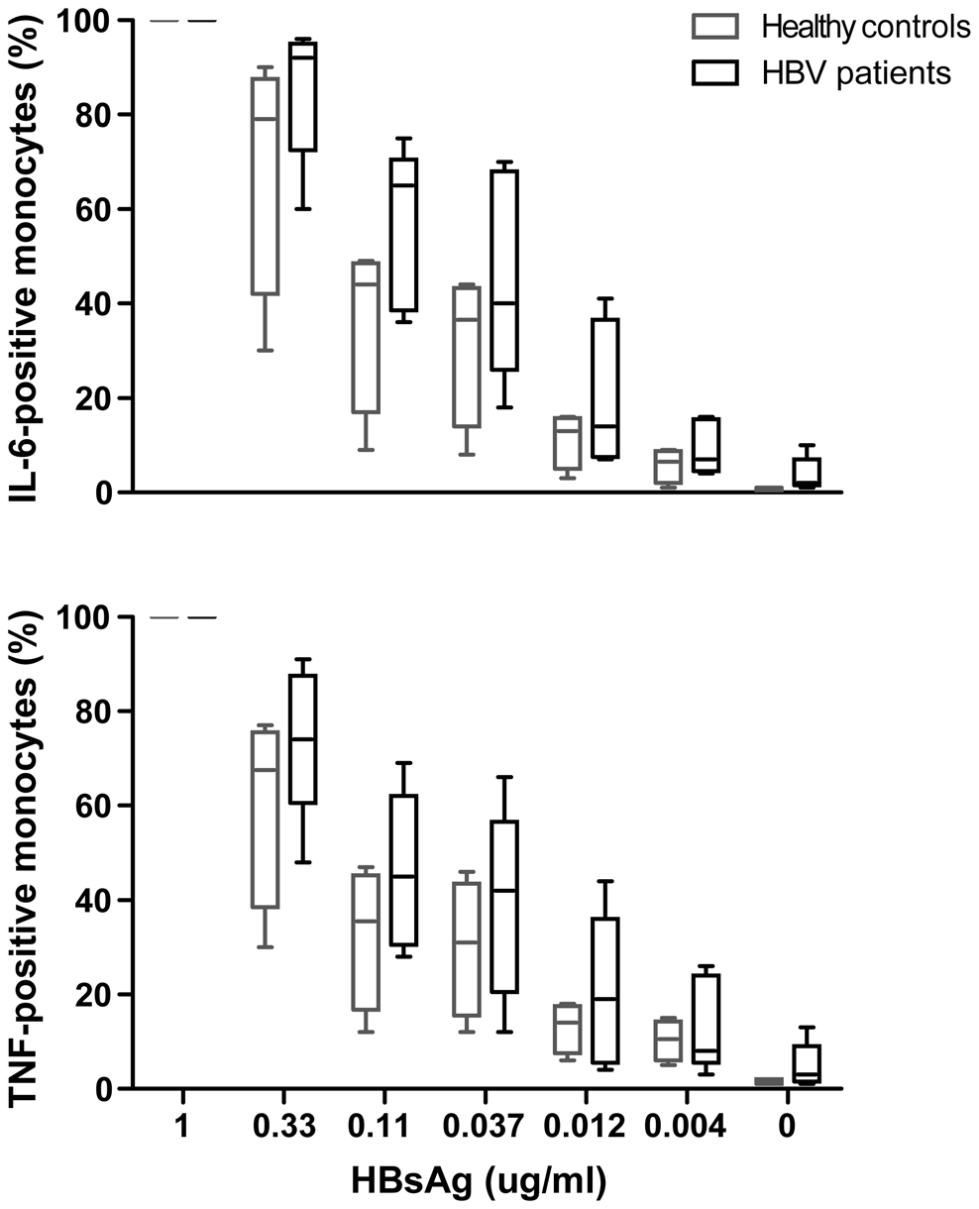
Monocytes from patients and healthy controls are equally sensitive to HBsAg *in vitro*. PBMC from healthy individuals or HBV patients with distinct *in vivo* exposure histories to HBV DNA, HBeAg and HBsAg (group 3) were stimulated with HBsAg and the relative frequency of cytokine-producing monocytes was determined (n = 5–6). In the box-whisker plots, the line in the middle of the box is the median, while the whiskers depict the minimum and the maximum value.

In contrast to the *in vitro* findings where exposure of monocytes to HBsAg leads to high cytokine induction, comparison of circulating monocytes obtained from patients with different levels of viral proteins *in vivo*, showed no higher spontaneous or TLR-induced cytokine induction. One possible explanation could be that continuous exposure of patient's leukocytes to HBV proteins makes them less sensitive to re-exposure. To examine this, we compared the *in vitro* effects of HBsAg on monocytes obtained from chronic HBV patients and from age- and sex-matched healthy individuals. Exposure of PBMC from patients to HBsAg resulted in IL-6 and TNF production, depicted by IL-6-positive (86.1±2.0%) and TNF-positive (62.1±6.6%) CD14^+^ monocytes, while also monocytes from healthy individuals were positive for IL-6 (78.2±5.4%) and TNF (55.4±9.0%). Next, to examine whether monocytes from patients and controls were equally sensitive to the stimulatory effect of HBsAg, cells were stimulated with a dose-range of the HBsAg protein. As shown in Figure 5, monocytes from healthy controls and HBV patients were equally sensitive to HBsAg at low, intermediate and high doses of HBsAg, and consequently the responsiveness of monocytes to overnight HBsAg stimulation was similar between both groups. Likewise, HBsAg stimulation of PBMC for 5 hours, instead of 18 hours, also showed similar frequencies of IL-6 and TNF-producing monocytes between healthy controls and HBV patients, as shown in Figure S1. This supplementary figure shows not only stimulation with pHBsAg Ay as used throughout the paper, but also with recombinant HBsAg, both resulting in no difference between the groups. Also, assessment of overnight cytokine production by ELISA confirmed that monocytes from healthy individuals and from HBV patients react identical to HBsAg in terms of cytokine production (data not shown).

### IL-10 *in vivo* potently inhibits HBsAg-induced cytokine production by monocytes

**Figure 6:**
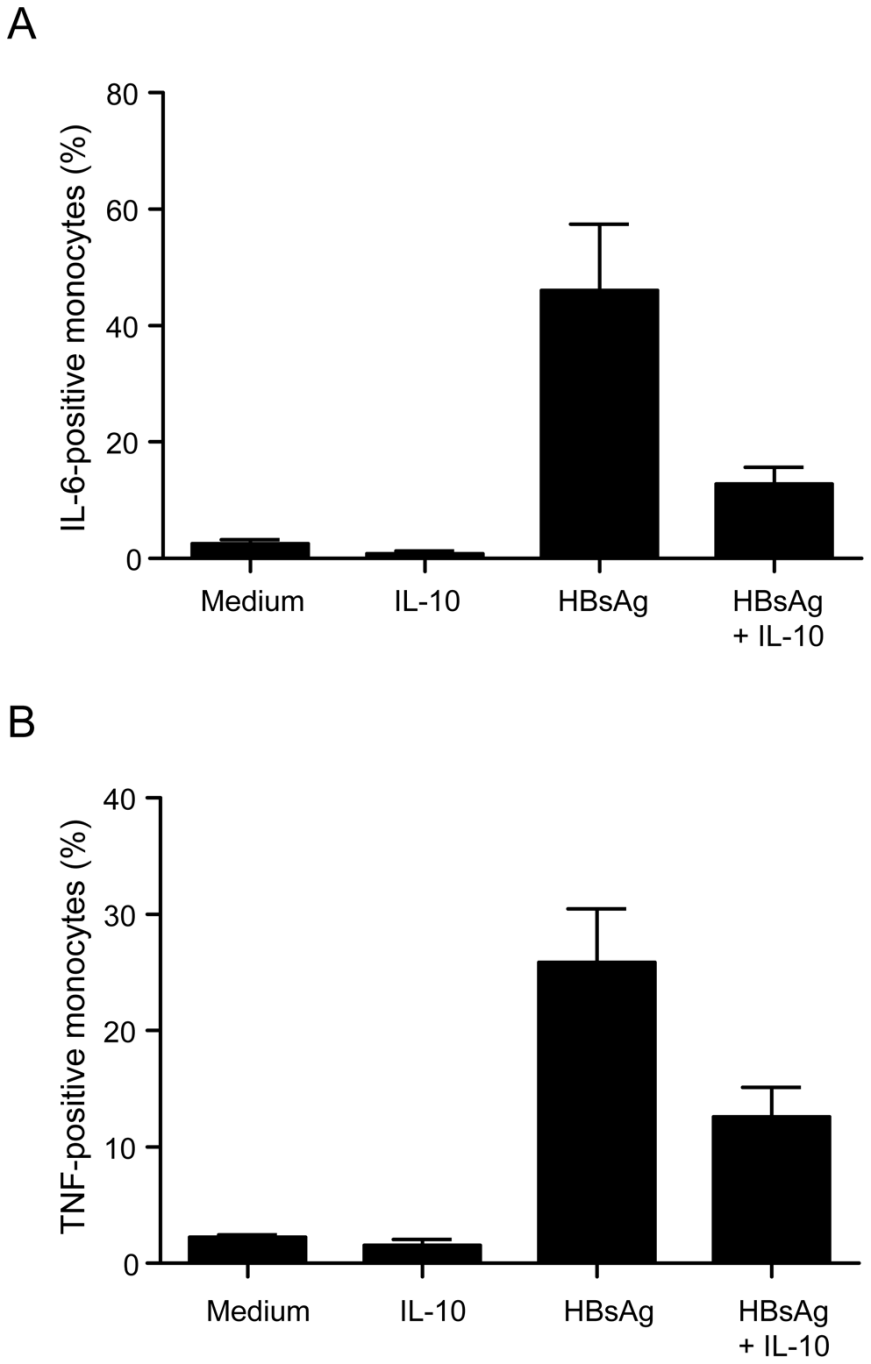
IL-10 inhibits the frequency of monocytes producing cytokines upon HBsAg exposure. PBMC from healthy individuals were stimulated with HBsAg, with or without IL-10. After overnight incubation, cells were stained for CD14 and intracellularly for cytokines. The frequencies of IL-6-positive (**A**) and TNF-positive (**B**) monocytes were compared between HBsAg and HBsAg and IL10 (n = 3).

Having demonstrated that monocytes from chronic HBV patients and healthy individuals are equally sensitive to HBsAg, we considered the activity of immunosuppressive cytokines, such as IL-10. In chronic HBV patients increased serum IL-10 levels have been demonstrated by various research groups [18]–[22]. Enhanced production of this immunosuppressive cytokine and increased sensitivity of activated monocytes to IL-10 may prevent monocyte activation by HBsAg *in vivo* [23], [24]. As depicted in Figure 6, the presence of IL-10 clearly abrogated HBsAg-induced cytokine production, demonstrating a possible *in vivo* mechanism to curb HBsAg-induced cytokine production by monocytes.

## Discussion

In the present study, we demonstrate that HBsAg has a potent stimulatory effect on human monocytes upon *in vitro* culture. However, detailed comparison of monocytes obtained from chronic HBV patients showed that the differences in viral load, and levels of viral proteins did not influence the functionality of the monocyte compartment, suggesting that mechanisms are in place to prevent *in vivo* activation of monocytes by viral proteins such as HBsAg.

The majority of chronic HBV patients who are not being treated have relatively high levels of HBsAg in serum. We showed that monocytes are responsive to HBsAg, and get activated as a consequence of *in vitro* exposure to HBsAg as evidenced by the release of IL-6 and TNF. In agreement with our observations, others have demonstrated that patient-derived HBsAg induced the production of TNF and IL-10 by monocytes [16], and that monocyte-derived DC produced IL-12 upon stimulation with HBsAg [25]. However, there is controversy on the effect of HBsAg, since it was also reported that HBV, HBeAg and HBsAg are inhibitory and can in some cases suppress TLR-induced cytokine production in various cell types [26]–[30], including monocytes *in vitro* [31] and *in vivo* [13]. Importantly, using the same preparation of HBsAg, we previously demonstrated suppression of TLR9-induced IFN-α by plasmacytoid DC as a consequence of exposure to HBsAg [29]. Furthermore, to rule out that the stimulatory effects on monocytes were unique for the preparation, we tested different preparations from different suppliers and showed that the stimulatory capacity of HBsAg on monocytes was a general feature (rHBsAg (Prospec, Rehovot, Israel), pHBsAg Adr, pHBsAg Ayw (both Jena Bioscience GmbH, Jena, Germany), data not shown). This could mean that monocytes contribute to the initiation phase of an anti-viral immune response against HBV, being able to recognize the viral envelope protein and produce pro-inflammatory cytokines in the periphery, and possibly also in the liver during inflammation.

Since the HBsAg concentrations that were used for *in vitro* stimulations are in the same range as found in the serum of chronic HBV patients (5 µg/ml HBsAg corresponding to ±22,000 IU/ml HBsAg) [32], and since monocytes have been shown to interact *in vivo* with HBsAg [33], one might expect that this has a profound effect on the monocyte compartment in patients. Consequently, it is expected that patients with distinct levels of serum HBsAg, but also of HBV DNA and HBeAg, demonstrate an altered monocyte phenotype and function. Our findings show that the distribution of CD14^++^CD16^-^ and CD14^+^CD16^+^ monocytes was not different between patients with distinctive virological characteristics. Our findings appear not be in agreement with a study by Zhang et al., which showed increased numbers of CD16^+^ monocytes in immune-active HBeAg-positive patients as compared to healthy individuals or immunotolerant patients [12]. It should be noted that monocyte subset frequencies correlate with ALT values in chronic HBV patients [12], and that the study by Zhang et al, and ours differ considerably with respect to the ALT ranges included (13-1656 versus 15–443 IU/L). Therefore, differences in inclusion criteria of patients to address the respective research questions may likely explain the results. Additionally, we showed that also the functionality, as demonstrated by the frequency of TLR-induced cytokine producing monocytes and the TLR-induced cytokine production in PBMC was comparable in chronic HBV patients with different levels of viral DNA and viral proteins. This important finding on limited modulation of the monocyte compartment in chronic HBV patients *in vivo* is not reflected by the immunostimulatory effect of HBsAg on monocytes *in vitro*, and clearly demonstrates that comparison of the *in vivo* and *in vitro* findings is complex. It is also important to note that reported findings on the effects of HBsAg on immune cells use other cell types or cell lines than primary monocytes and different recombinant or plasma-derived preparations of HBsAg, which complicates the interpretation of the *in vitro* findings [26]-[31], [34]. Our findings are in line with Gehring et al., who showed that despite a constant exposure to HBsAg, *ex vivo*–isolated monocytes did not constitutively activate HBV-specific CD8^+^ T cells [33] Evaluation of monocytes from chronic HBV patients showed that the induction of cytokines upon TLR ligation was not affected by the clinical phase. Furthermore, the spontaneous cytokine release of these monocytes was similar between all groups, suggesting that the continuous exposure to variable concentrations of HBV DNA, HBeAg or HBsAg did not result in modulation of the *ex vivo* activation state of these cells.

Combined these findings suggest that the monocyte compartment of patients is not affected as a consequence of exposure to different concentrations of viral proteins and HBV DNA in serum. Since this is in apparent conflict with the *in vitro* experiments in which monocytes were exposed to HBsAg, it is tempting to speculate that regulatory and/or compensatory mechanisms are in place *in vivo*, which prevent excessive activation of monocytes by serum HBsAg. We demonstrated that it is unlikely that unresponsiveness of patients' monocytes to HBsAg due to desensitization as a consequence of recent exposure to the same protein may explain the discrepancy between the *in vitro* and *in vivo* observations, since monocytes from patients are similarly responsive to HBsAg as monocytes from healthy individuals, as shown by dose titrations. However, a likely candidate that may contribute to explain the *in vivo* data is the immunosuppressive cytokine IL-10 since it has been reported that chronic HBV patients have increased serum IL-10 levels [18]–[22]. Our data indeed show that IL-10 is able to inhibit cytokine production by HBsAg-induced monocytes, suggesting a possible role of IL-10 in restraining pro-inflammatory cytokine production by monocytes in HBV patients with high levels of HBsAg, HBeAg and HBV DNA. However, also other candidate molecules present in serum, such as TGF-β may further regulate monocyte function in chronic HBV patients.

In summary, we demonstrate that HBsAg has potent stimulatory effects on monocytes *in vitro*. However, in chronic HBV patients, the functionality of the monocyte compartment is not influenced by variations in the levels of serum viral components, suggesting that regulatory mechanisms are in place to prevent excessive *in vivo* activation of monocytes.

## Supporting Information

Figure S1 **Monocytes from patients and healthy controls are equally sensitive to HBsAg** ***in vitro*** **.** PBMC from healthy individuals or HBV patients with distinct *in vivo* exposure histories to HBV DNA, HBeAg and HBsAg (group 3) were stimulated for 5 hours with patient plasma-derived HBsAg or recombinant HBsAg and the frequency of cytokine-producing monocytes was determined (n = 5–6). In the box-whisker plots, the line in the middle of the box is the median, while the whiskers depict the minimum and the maximum value.
(TIF)
