# Supplementary figures and images for: Monocytes from Chronic HBV Patients React In Vitro to HBsAg and TLR by Producing Cytokines Irrespective of Stage of Disease
Source: PLoS One. 2014 May 13;9(5):e97006. doi: 10.1371/journal.pone.0097006 (PMC4019549; doi:10.1371/journal.pone.0097006)

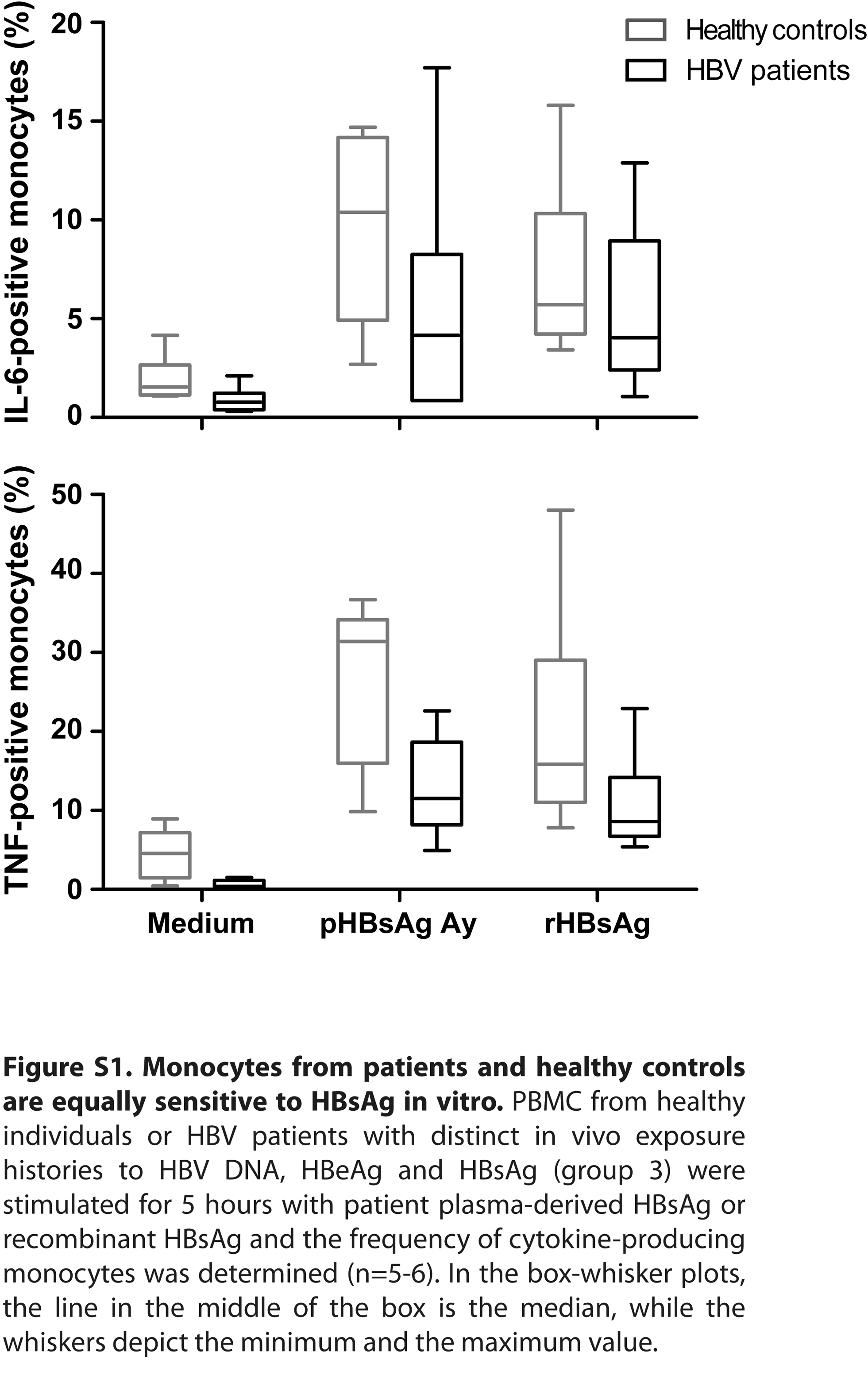

Supplement: Figure S1 — Monocytes from patients and healthy controls are equally sensitive to HBsAg in vitro . PBMC from healthy individuals or HBV patients with distinct in vivo exposure histories to HBV DNA, HBeAg and HBsAg (group 3) were stimulated for 5 hours with patient plasma-derived HBsAg or recombinant HBsAg and the frequency of cytokine-producing monocytes was determined (n = 5–6). In the box-whisker plots, the line in the middle of the box is the median, while the whiskers depict the minimum and the maximum value. (TIF) [file pone.0097006.s001.tif]
